# Understanding Electro-communication and Electro-sensing in Weakly Electric Fish using Multi-Agent Deep Reinforcement Learning

**Published:** 2025-11-11

**Authors:** Satpreet H. Singh, Sonja Johnson-Yu, Zhouyang Lu, Aaron Walsman, Federico Pedraja, Denis Turcu, Pratyusha Sharma, Naomi Saphra, Nathaniel B. Sawtell, Kanaka Rajan

**Affiliations:** Harvard University; Harvard University; Brown University; Harvard University; Columbia University; Columbia University; MIT; Harvard University; Columbia University; Harvard University

## Abstract

Weakly electric fish, like *Gnathonemus petersii*, use a remarkable electrical modality for active sensing and communication, but studying their rich electrosensing and electrocommunication behavior and associated neural activity in naturalistic settings remains experimentally challenging. Here, we present a novel biologically-inspired computational framework to study these behaviors, where recurrent neural network (RNN) based *artificial agents* trained via multi-agent reinforcement learning (MARL) learn to modulate their electric organ discharges (EODs) and movement patterns to collectively forage in virtual environments. Trained agents demonstrate several emergent features consistent with real fish collectives, including heavy tailed EOD interval distributions, environmental context dependent shifts in EOD interval distributions, and social interaction patterns like *freeloading*, where agents reduce their EOD rates while benefiting from neighboring agents’ active sensing. A minimal two-fish assay further isolates the role of electrocommunication, showing that access to conspecific EODs and relative dominance jointly shape foraging success. Notably, these behaviors emerge through evolution-inspired rewards for individual fitness and emergent inter-agent interactions, rather than through rewarding agents explicitly for social interactions. Our work has broad implications for the neuroethology of weakly electric fish, as well as other social, communicating animals in which extensive recordings from multiple individuals, and thus traditional data-driven modeling, are infeasible.

## 1 Introduction

Communication signals in non-human animals are rich, context-dependent, and often difficult to study systematically in their natural habitat. Weakly electric fish provide a striking example: they use electric organ discharges (EODs) both for active electrosensing and for social communication [1, 2, 3, 4]. EODs encode information about dominance, foraging, and collective behavior, Experimental study of these signals is challenging because it requires recording from multiple freely moving individuals in naturalistic settings. Such complexities have limited our ability to build models of what, exactly, EODs communicate.

Multi-agent reinforcement learning (MARL) offers an opportunity to address this gap, and has been previously applied to model collective behaviors such as flocking and cooperative hunting [5, 6, 7]. Here we adapt MARL to explicitly study electro-communication and electrosensing. We build recurrent neural network (RNN) agents that emit and sense EODs in a simulated environment, with biologically inspired constraints on movement, electric field generation, electrosensory receptors, and long-range social sensing [8, 2]. Trained agents reproduce key empirical phenomena: heavy-tailed EOD interval statistics, social “freeloading” strategies, and stereotyped pairwise motifs. A minimal two-fish assay further isolates the role of communication, showing that access to conspecific EODs strongly shapes foraging outcomes.

Crucially, our framework provides complete access to the internal dynamics of the RNN agents. This enables mechanistic interpretability—linking specific sensory inputs to hidden representations and EOD outputs—as well as targeted ablation and steering experiments. In this way, the model serves not only as a testbed for hypotheses about fish behavior, but also as a generator of synthetic communication corpora that can be analyzed with tools from unsupervised machine translation and related areas [9, 10]. Our goal is to bridge AI and animal communication by developing controllable, interpretable models that help uncover the functional role of non-human communication signals.

## 2 Methods

Our framework couples a custom 2D physics simulator with multi-agent reinforcement learning (MARL), where artificial agents interact to forage for food, mimicking a typical experimental setup. We implement patchy food distributions with variable replenishment rates to simulate both competitive (zero-sum) and non-competitive foraging scenarios. The simulator manages agent kinematics, food dynamics, and the generation and propagation of electric fields.

Agents act in continuous space by selecting forward movement, turning, binary electric organ discharges (EODs) and biting conspecifics. Each emitted EOD produces an electric field that interacts with food, walls, and conspecifics, generating induced sources and reflections that are then transduced into egocentric observations. Agents sense this electric landscape through a compact set of biomimetic sensor channels that capture (i) distortions of their own EODs (short-range active sensing), (ii) low-frequency background fields (passive sensing), and (iii) sharp EOD pulses from conspecifics (long-range *social* sensing) [12]. The framework also models how agents can use the EODs of neighbors to sense their environment, a phenomenon called *collective sensing* [4].

**Figure 1:**
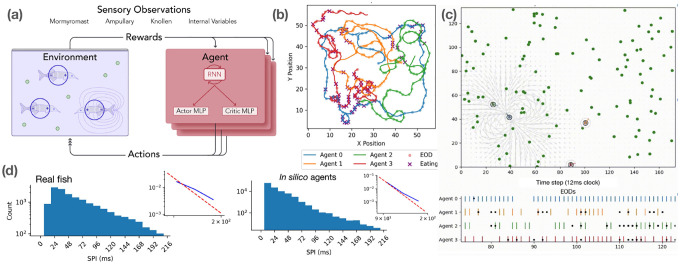
Overview of our MARL framework for modeling weakly electric fish communication. **(a)** Schematic of the training loop, where agents interact with a simulated arena, emitting and sensing electric organ discharges (EODs) through weakly electric fish-inspired sensors. Rewards encourage successful foraging and penalize aggressive encounters. **(b)** Example trajectories from four agents in a single foraging episode, showing exploration and food acquisition. **(c)** Snapshot of the arena (top) showing agents, food sources, and simulated electric fields; bottom shows temporally-structured EOD spike trains across individual agents. **(d)** Sequential Pulse Interval (SPI) distributions from real fish (left) and MARL-trained agents (right), showing that in silico agents reproduce the heavy-tailed statistics observed in biological data. Insets show log-linear curves compared to empirical curve fits.

Agent share individual instantiations of a common underlying neural network consisting of a recurrent neural network (RNN) followed by parallel Actor and Critic two-layer feedforward networks (MLPs) (Figure 1a). All layers are 512 units wide, with *tanh* nonlinearities. Agents are trained using Multi-Agent Proximal Policy Optimization [13, 14, 15] with rewards that encourage successful foraging and provide asymmetric penalties during aggressive encounters between fish of different dominance levels. Trained MARL agents develop socially aware foraging strategies, dominance displays, and context-dependent EOD modulation. Importantly, no explicit collective behaviors are rewarded, coordination and communication emerge solely from individual fitness optimization in a shared environment.

In summary, our design yields agents that can both forage effectively and develop communication-like behaviors, while also exposing full access to their internal RNN dynamics for interpretability, ablation, and steering analyses (Figure 1).

## 3 Analyses

**Figure 2:**
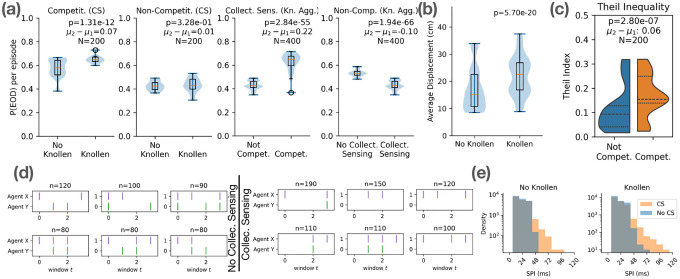
**(a)** Comparison of EOD probabilities under various conditions: (Left to Right) **(a1)** Effect of the Knollenorgan in competitive environments. The presence of the Knollenorgan (which provides long-range information about other agents) increases EOD rates in competitive scenarios only, suggesting the importance of social information in limited-resource regimes. **(a2)** Effect of the Knollenorgan in non-competitive environments. The Knollenorgan has no impact on EOD rates in non-competitive scenarios, suggesting that long-range information is not important when food is abundant. **(a3)** Agents (with collective sensing and Knollenorgan-enabled long-range sensing) generally tend to produce more EODs in arenas with limited food (“Competition”) compared to cases where food supply is unlimited (“No competition”), suggesting that competition drives higher EOD rates as agents actively search for food. **(a4)** Effect of collective sensing in non-competitive environments, aggregated with and without Knollenorgan-based long-range sensing. Collective sensing reduces EOD rates as agents can gather short-range information from the EOD discharges of their neighbors, consistent with [4]. **(b)** Agent displacement over a ≈ 0.36 second window in non-competitive environments indicates that long-range social information associated with larger movement bouts, potentially facilitating more extensive spatial exploration and thereby more efficient foraging. **(c)** Inter-agent inequality in food consumption increases under food scarcity, as measured by Theil Index [11]. **(d)** Top 6 most common pairwise **social EOD motifs** (Left) without collective sensing in non-competitive environments, vs. (Right) with collective sensing enabled. Higher occurrence of “silent” periods in one agent reveals emergent “freeloading” behavior during collective sensing. Social EOD motifs defined here as interaction between two agents within 15 cm for at least 4 timesteps. **(e)** SPI distributions shift upwards (equivalently, EOD rates lower) during collective sensing (CS) further supporting the emergence of “freeloading” strategies.

**Figure 3:**
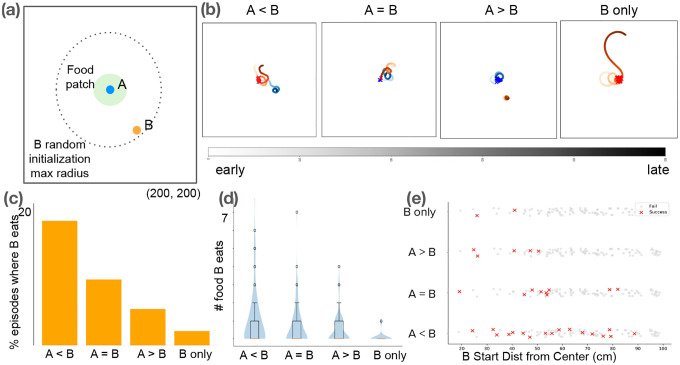
**(a)** Minimal social foraging assay with two agents, A and B. A is initialized within a fully-replenishing food patch, while B is randomly initialized within communication radius to A. **(b)** Example trajectories in different A/B relative dominance scenarios. **(c)** We vary the relative dominance levels of A/B, then compare the percentage of trials where B reaches the patch (100 runs). B performs better when it is more dominant. However, B’s performance drops dramatically when agent A is removed, suggesting that there is a social component to foraging success. **(d)** Amount of food eaten by B per episode follows similar trends w.r.t. dominance. **(e)** B’s success is modulated by both dominance and starting location, indicating a social component to spatial foraging strategy [4].

Our analyses show that trained agents learn to forage effectively (Fig. 1b,c) while developing emergent electro-sensing and electro-communication strategies that parallel experimental observations. EOD interval distributions reproduce the heavy-tailed statistics of real fish (Fig. 1d) and are strongly modulated by environmental context (Fig. 2a). Conspecific presence enables “freeloading,” where agents reduce their own discharges while exploiting neighbors’ activity (Fig. 2a,d,e) [16, 17]. At the group level, “social sensors” promote longer-range movements (Fig. 2b), and food scarcity amplifies inequality in consumption (Fig. 2c). Finally, a minimal two-fish assay (Fig. 3) isolates the social-communication component, demonstrating that access to conspecific EODs encourages foraging success and approach strategy, and is heavily modulated by inter-agent dominance differences.

## 4 Discussion

Our work demonstrates the potential of multi-agent reinforcement learning to complement experimental studies of naturalistic behavior in weakly electric fish, using *in silico* experiments and analyses. Since our framework provides full control over agent dynamics, it allows targeted steering and ablation experiments to isolate what specific EOD patterns communicate. Future work will extend this approach with richer ecological contexts (e.g., predators, sex differences) and with methods from unsupervised machine translation [18, 19, 20] to directly test whether emergent signal repertoires can be aligned with experimental datasets. We also plan to perform additional in silico experiments, mechanistic interpretation of the RNNs [21, 22, 23, 24], and steering experiments that connect emergent signals to functional outcomes [9, 10]. Ultimately, we intend to use our model to generate specific, testable hypotheses about electric fish communication in natural and controlled settings, guiding future experimental studies [25].

### Ethical considerations:

Our framework reduces invasive experimentation on live animals by enabling in silico hypothesis generation and prioritization. All experimental data used in this paper were collected by collaborators for previous neuroscientific studies of weakly electric fish [4, 26].
